# Antimicrobial Activity of Nanoconjugated Glycopeptide Antibiotics and Their Effect on *Staphylococcus aureus* Biofilm

**DOI:** 10.3389/fmicb.2021.657431

**Published:** 2021-12-02

**Authors:** Francesca Berini, Viviana Teresa Orlandi, Federica Gamberoni, Eleonora Martegani, Ilaria Armenia, Rosalba Gornati, Giovanni Bernardini, Flavia Marinelli

**Affiliations:** ^1^Department of Biotechnology and Life Sciences, University of Insubria, Varese, Italy; ^2^Instituto de Nanociencia y Materiales de Aragón (INMA), CSIC-Universidad de Zaragoza, Zaragoza, Spain

**Keywords:** magnetic nanoparticles, iron oxide nanoparticles, teicoplanin, vancomycin, antimicrobial resistance, biofilm

## Abstract

In the era of antimicrobial resistance, the use of nanoconjugated antibiotics is regarded as a promising approach for preventing and fighting infections caused by resistant bacteria, including those exacerbated by the formation of difficult-to-treat bacterial biofilms. Thanks to their biocompatibility and magnetic properties, iron oxide nanoparticles (IONPs) are particularly attractive as antibiotic carriers for the targeting therapy. IONPs can direct conjugated antibiotics to infection sites by the use of an external magnet, facilitating tissue penetration and disturbing biofilm formation. As a consequence of antibiotic localization, a decrease in its administration dosage might be possible, reducing the side effects to non-targeted organs and the risk of antibiotic resistance spread in the commensal microbiota. Here, we prepared nanoformulations of the ‘last-resort’ glycopeptides teicoplanin and vancomycin by conjugating them to IONPs *via* surface functionalization with (3-aminopropyl) triethoxysilane (APTES). These superparamagnetic NP-TEICO and NP-VANCO were chemically stable and NP-TEICO (better than NP-VANCO) conserved the typical spectrum of antimicrobial activity of glycopeptide antibiotics, being effective against a panel of staphylococci and enterococci, including clinical isolates and resistant strains. By a combination of different methodological approaches, we proved that NP-TEICO and, although to a lesser extent, NP-VANCO were effective in reducing biofilm formation by three methicillin-sensitive or resistant *Staphylococcus aureus* strains. Moreover, when attracted and concentrated by the action of an external magnet, NP-TEICO exerted a localized inhibitory effect on *S. aureus* biofilm formation at low antibiotic concentration. Finally, we proved that the conjugation of glycopeptide antibiotics to IONPs reduced their intrinsic cytotoxicity toward a human cell line.

## Introduction

The spread of antibiotic-resistant bacteria, exacerbated by the inappropriate use and abuse of antibiotics, is considered one of the main causes of morbidity and mortality worldwide, resulting in an increasing economic burden for the health systems (Cassini et al., 2018). Currently, drug-resistant bacteria cause 700,000 deaths per year and, according to the World Health Organization (WHO), by 2050, this number will reach 10 million deaths per year if no action is taken (Inter-agency Coordination Group on Antimicrobial Resistance, 2019). Nowadays, the COVID-19 pandemic has further complicated the era of antimicrobial resistance (AMR), since most of the patients with COVID-19 are treated for presumptive or confirmed secondary infections by broad spectrum antimicrobials (Nieuwlaat et al., 2020). One of the most common and difficult-to-treat drug-resistant infection is due to *Staphylococcus aureus*, whose incidence in hospital-acquired infections is very high. Indeed, recent data indicate that, in the WHO member states, from 20 to 80% of *S. aureus* clinical isolates are resistant to methicillin. Unfortunately, some of these isolates are also resistant to other antibiotics, including the last-resort carbapenems, glycopeptides, and more recently daptomycin (Gao et al., 2020; Shariati et al., 2020). Additionally, methicillin susceptible *S. aureus* (MSSA) or methicillin-resistant (MRSA) strains produce biofilms, which play a crucial role in establishing and sustaining difficult-to-treat severe infections such as osteomyelitis, endocarditis, and chronic bound infections (Uruén et al., 2020). Biofilms consist of organized layers of bacteria cells attached to a surface and embedded in an extracellular polymeric matrix, which is a complex and variable biochemical mixture of polysaccharides, proteins, glycopeptides, nucleic acids, and lipids. Cells, once embedded in the biofilm, are protected from the host immunological response and become up to 1,000 times less susceptible to antibiotics than their planktonic mobile counterparts (Arciola et al., 2018; Fulaz et al., 2020). Medical devices, such as intravenous and urinary catheters, vascular, heart valve and joint prostheses, pacemakers, and contact lenses, provide excellent surfaces for *S. aureus* biofilm formation contributing to the diffusion and persistence of resistant infections (Arciola et al., 2018).

Despite the burden of AMR, the number of novel antibiotic classes actually introduced into the market since 2000 is worryingly low. This is due to a multiplicity of factors, including difficulties in isolating novel molecules able to counteract the emerging resistance mechanisms and to eradicate biofilms, and a series of regulatory and economical constrains that affect antibiotic marketplace and discourage investments by pharma companies (Renwick and Mossialos, 2018). An alternative to the *de novo* antibiotic discovery, which is a long and costly process, is the development of diverse antimicrobial treatments by exploring less conventional solutions (i.e., using bacteriophages, antibacterial antibodies and peptides, photodynamic therapy) and by repurposing existing drugs using innovative formulations and administration routes (i.e., combining antibiotics with adjuvants or AMR inhibitors, liposomes, and nanomaterial for encapsulation and targeting; Mulani et al., 2019; Naskar and Kim, 2019).

Nanomaterials can be used as active therapeutic agents *per se*, or as carriers for already known antimicrobial molecules, or both (Gupta et al., 2019). Silver, copper, copper oxide, zinc oxide, titanium oxide, and chitosan nanoparticles (NPs) are examples of nanomaterials with proven anti-microbial characteristics due to variegate and often not yet-completely understood mechanisms of actions (Joshi et al., 2020; Wang et al., 2020). Metal oxide NPs, in particular, are considered promising in penetrating tissues and disturbing biofilm structures, due to their physicochemical characteristics as small size, large surface-area-to-volume ratio, crystalline structure with a number of edges and corners and reactive sites, and, importantly, their potential to establish electrostatic interactions between their positively charged metal ions with the negatively charged biofilm matrix and bacteria cell envelopes (Al-Shabib et al., 2018; Joshi et al., 2020; Shkodenko et al., 2020). Iron oxide NPs (IONPs) are a special class of metal oxide NPs possessing superparamagnetic properties (Ajinkya et al., 2020). IONPs are already in use for diagnostic imaging (magnetic resonance imaging, MRI), cancer treatments (magnetic hyperthermia, thermal ablation), scaling up bioseparation processes and biosensing-based applications (Ajinkya et al., 2020), and they are promising for remotely controlled nanoactuation of enzyme activity (Armenia et al., 2019). As antibiotic carriers, IONPs offer the potential of specifically directing the active principles to the site of infection and/or conveying them deep into the biofilms by simply using an external magnetic field (Arias et al., 2018; Shkodenko et al., 2020). As a consequence, the efficacy of the localized antimicrobial treatment might increase, thus allowing the administration of lower doses with a consequent minimization of the intrinsic toxicity of the antibiotic to non-targeted and healthy organs and of systemic side effects. Moreover, the exposure of commensal microbiota to sub-lethal doses of the antibiotic might be reduced, thus preventing the rise of AMR. Clinical applications of magnetic IONPs are also supported by their physical and chemical stability and superior biocompatibility in comparison to other metal oxide NPs (Arias et al., 2018; Shkodenko et al., 2020) as well as by their relative safety (Bonfanti et al., 2020).

In this paper, we explored IONPs as carriers for two drugs of last resort, the glycopeptide antibiotics (GPAs) vancomycin and teicoplanin, currently in clinical use for treating severe infections caused by Gram-positive pathogens, including endocarditis, meningitis, and complicated skin, bloodstream, bone, and joint infections. Indeed, these GPAs, despite being relatively old antibiotics (with vancomycin approved by the Food and Drug Administration in the 1950s, and teicoplanin introduced in the European market in the 1980s), still play a key role in mainstream therapy against MRSA and other clinically relevant Gram-positive pathogens (Butler et al., 2014; Marcone et al., 2018). Vancomycin and teicoplanin are natural products, synthesized by actinomycetes and formed by a nonribosomal heptapeptide scaffold, decorated with sugar moieties, chlorine atoms, methyl groups, and, in the case of teicoplanin, a lipid chain (Yushchuk et al., 2020a). They exert their antibacterial activity by inhibiting cell wall synthesis, through binding to the d-alanyl-d-alanine terminus of the peptidoglycan precursor, thus leading to cell death (Binda et al., 2014). Dose-dependent nephrotoxicity (more severe in the case of vancomycin) and poor penetration in biofilms and into certain body tissues are nowadays recognized as weak points in GPA therapeutic use (Van Bambeke, 2006; Gao et al., 2020). Moreover, the spread of GPA-resistance, first among enterococci and then in staphylococci, has led WHO to include GPAs among the “watch group” of the Essential Medicine List, i.e., among those antimicrobials that should be prescribed only for specific indications and subjected to stewardship programs and monitoring to avoid diffusion of resistant isolates (Sharland et al., 2018). All these factors encourage searching and developing alternative formulations that might improve GPA delivery and efficacy, while keeping toxicity and resistance under control.

In the course of the past 10years, different authors have investigated the possibility to incorporate vancomycin into several nanomaterials such as liposomes, chitosan scaffolds, or gold nanostars (Uhl et al., 2017; Gonzalez Gomez et al., 2019; Karakeçili et al., 2019; Wang et al., 2019). Indeed, to the best of our knowledge, only two papers have explored the potential of vancomycin conjugation to IONPs, the first one for developing an affinity capture system for Gram-positive and Gram-negative bacteria in biological samples (Kell et al., 2008) and the more recent one for enhancing vancomycin sporicidal efficacy against *Clostridium difficile* (Chen et al., 2019). Less investigated has been the generation of nanosystems based on teicoplanin. The antimicrobial potential of nanocarried teicoplanin was reported only by Peng et al. (2010), who treated osteomyelitis with teicoplanin-encapsulated biodegradable thermosensitive hydrogel; by Gonzalez Gomez et al. (2019), who tested liposome-encapsulated teicoplanin towards *S. aureus*; and by Ucak et al. (2020), who reported teicoplanin delivery in poly lactic-co-glycolic acid NPs functionalized with *S. aureus* specific aptamers. In 2018, our group succeeded for the first time in conjugating teicoplanin to magnetic IONPs, after their functionalization with (3-aminopropyl) triethoxysilane (APTES; Armenia et al., 2018). In the present paper, we have investigated the synthesis and the efficacy of IONPs carrying vancomycin and teicoplanin, as an innovative system for GPA formulation and use. We have paid particular attention to the effect of the two nanoconjugated antibiotics in inhibiting *S. aureus* biofilm formation.

## Materials and Methods

### Materials

Acetonitrile (CH_3_CN), ammonium formate (HCOONH_4_), ammonium hydroxide (NH_4_OH), (3-aminopropyl) triethoxysilane (APTES), bis(sulfosuccinimidyl) suberate (BS3), boric acid (H_3_BO_3_), crystal violet (C_25_N_3_H_30_Cl), 2′,7′-dichlorodihydrofluorescein (DCFH-DA), 4,4-difluoro-1,3,5,7-tetramethyl-8(2′methoxyphenyl)-4-bora-3a,4a-diaza-s-indacene, ethanol (C_2_H_6_O), ferric nitrate (Fe(NO_3_)_3_×9 H_2_O), fetal bovine serum (FBS), formaldehyde (CH_2_O), glutamine (C_5_H_10_N_2_O_3_), glutaraldehyde (C_5_H_8_O_2_), hydrochloric acid (HCl), iron dichloride (FeCl_2_×4 H_2_O), iron trichloride (FeCl_3_×6 H_2_O), Luria Bertani broth (LB), Luria Bertani agar (LB agar), 2-(N-morpholino) ethanesulfonic acid (MES), Müller Hinton agar (MHA), Müller Hinton broth 2 (MHB2), nitric acid (HNO_3_), osmium tetroxide (OsO_4_), phorbol 12-myristate 13-acetate (PMA), phosphate-buffered saline (PBS), potassium ferrocyanide (K_4_[Fe(CN)_6_]×3H_2_O), RPMI-1640 medium, sodium chloride (NaCl), sodium hydroxide (NaOH), teicoplanin, tryptic soy broth (TSB), and vancomycin were purchased from Sigma-Aldrich, Milan, Italy. All chemical reagents were used without additional purification.

### Microbial Strains and Culture Conditions

*Escherichia coli* ATCC 35218, *Bacillus subtilis* ATCC 6633, *Staphylococcus aureus* ATCC 6538P (MSSA), *Staphylococcus aureus* ATCC 25923 (MSSA), *Staphylococcus aureus* ATCC 43300 (MRSA), *Enterococcus faecalis* ATCC 29212, and *E. faecalis* ATCC 51299 (VanB phenotype) were obtained from the American Type Culture Collection (ATCC). *Enterococcus faecalis* 9160188401-EF-34 (VanA phenotype) and *Staphylococcus epidermidis* strain 4 are clinical isolates, kindly provided by Laboratorio Microbiologia Clinica – Ospedale di Circolo, Varese, Italy. *Staphylococcus haemolyticus* 3902 is a teicoplanin-resistant clinical isolate (Beltrametti et al., 2003), received from FIIRV (Fondazione Istituto Insubrico Ricerca per la Vita), Gerenzano Varese, Italy. For long-term preservation, bacterial cultures were stored at -80°C in 10% *v*/*v* glycerol.

*E. coli* and *B. subtilis* were routinely grown at 37°C with continuous shaking at 200rpm (revolutions per minute) in LB broth. *S. aureus*, *S. epidermidis*, *S. haemolyticus*, and *E. faecalis* strains were propagated in the same conditions in MHB2. For exponential growth, overnight cultures were diluted in fresh medium at an optical density at 600nm (OD_600nm_) of 0.1 and incubated as above.

### Synthesis of Nanoconjugated Antibiotics

Iron oxide (Fe_2_O_3_) NPs were synthetized using the co-precipitation method and functionalized with APTES, following the protocols described in Balzaretti et al. (2017) and Armenia et al. (2018). For conjugating vancomycin and teicoplanin, after having tried different combinations varying the GPAs and linker concentrations, the protocol used was the following. One milliliter of a 4mg/ml suspension of NP-APTES in 10mM borate buffer pH 8.2 was added with an amount of BS3 equal to 22.3μg (for conjugation with teicoplanin) or 44.6μg (in the case of vancomycin). The mixture was maintained under mechanical stirring for 1h at room temperature. Subsequently, 1mg of teicoplanin or 2mg of vancomycin was added to the NPs and the reaction allowed to proceed for 1h at 40°C under mechanical agitation. The reaction was stopped by adding 500μl of 10mM Tris-HCl at pH 8.0. Nanoconjugated teicoplanin (NP-TEICO) and nanoconjugated vancomycin (NP-VANCO) were isolated using a magnet, resuspended in 30mM MES buffer pH 6.0, and stored at 4°C. The amount of antibiotic bound to IONPs was estimated as follows:


conjugatedGPA=initialGPAadded to the reaction mixture−freeGPAmeasured in the supernatants afterNP-TEICO andNP-VANCO recoverybythe magnet.

The GPA concentration in the supernatant was calculated by UV detection at 280nm, using a UV-Vis JASCO V-460 spectrophotometer (Jasco, Easton, United States), and with the following linear regression equations: *y*=0.0045*x*+0.0373 (*R*^2^=0.9908) for teicoplanin; *y*=0.0038*x*+0.01046 (*R*^2^=0.9999) for vancomycin.

### NP Characterization

Shape, size, and size distribution of examined NPs were evaluated by transmission electron microscopy (TEM) using a JEOL 1010 electron microscope (JEOL, Tokyo, Japan). Samples were appropriately diluted in MilliQ water and dispersed on carbon-coated copper grids, then allowed to dry at room temperature. Hydrodynamic diameter and polydispersity index (PDI) were measured in MilliQ water at a concentration of 40μg/ml of NPs. Electrophoretic mobility (ζ-potential) measurements were performed on samples (at 2μg/ml final concentration) diluted in 1mM KCl, using a 90 Plus Particle Size Analyzer (Brookhaven Instrument Corporation, Holtsville, United States), operating at 25°C. The effective conjugation of APTES, vancomycin, and teicoplanin to IONPs was followed by Fourier Transform Infrared Spectroscopy in Attenuated Total Reflectance (FTIR-ATR), too. For this analysis, 10mg of the different NP preparations was dried in an oven at 50°C for 48h and, subsequently, FTIR-ATR analysis was carried out using an infrared spectrophotometer (Cary 630 FTIR; Agilent Technologies, Santa Clara, United States).

### Antimicrobial Susceptibility Test

Agar diffusion assay method was applied to evaluate the antimicrobial activity of NP-TEICO and NP-VANCO against *B. subtilis* ATCC 6633 and *S. aureus* ATCC 25923 (Armenia et al., 2018). Briefly, bacterial cultures were grown in MHB2 to an OD_600nm_ of 0.3–0.4 and then added at 10% *v*/*v* to MHA in Petri dishes. Ten microliter of IONPs, NP-TEICO, and NP-VANCO (at 4mg/ml concentration, loaded with 615μg/ml teicoplanin for NP-TEICO or 840μg/ml vancomycin for NP-VANCO) in 30mM MES buffer pH 6.0, as well as 10μl of teicoplanin and vancomycin in the same buffer (at a concentration equal to that loaded on the corresponding IONPs) was dropped onto the inoculated plates. Following incubation at 37°C for 24h, diameters of zones of bacterial growth inhibition surrounding the droplets were measured.

### Chemical Stability and Maintenance of the Antimicrobial Activity

NP-TEICO and NP-VANCO were stored at -20, 4, or 25°C up to 1month. Every 7days, samples were centrifuged at 14,700× *g* for 5min: the concentration of free antibiotics in the supernatants was quantified by spectrophotometric assay at 280nm and used to calculate the amounts of GPAs still bound to NPs. The antimicrobial activity of NP-TEICO and NP-VANCO was evaluated at the same time intervals by the antibiotic susceptibility test on *S. aureus* ATCC 25923.

### Determination of Minimum Inhibitory Concentrations and Minimal Bactericidal Concentrations

Minimum inhibitory concentrations (MICs) of nonconjugated and nanoconjugated teicoplanin and vancomycin towards *B. subtilis*, *S. aureus*, *S. epidermidis*, *S. haemolyticus*, *E. faecalis*, and *E. coli* strains were determined following the guidelines of the Clinical and Laboratory Standards Institute (CLSI, 2018), with broth dilution method in MHB2. Hence, *ca*. 5×10^5^ bacterial cells in exponential growth were inoculated into MHB2, supplemented with increasing concentrations of nonconjugated or nanoconjugated teicoplanin or vancomycin, dissolved in 30mM MES buffer pH 6.0, and incubated for 16–20h at 37°C and 200rpm. NP-TEICO and NP-VANCO concentrations to be added were calculated considering the amounts of teicoplanin or vancomycin loaded onto IONPs under the reaction conditions described above. MICs were expressed as the minimal concentrations of antibiotic at which no turbidity could be detected. For the determination of minimal bactericidal concentrations (MBCs), 100μl of bacterial cultures used for the MIC test were plated onto MHA, incubated at 37°C for 24h. MBCs were defined as the lowest concentrations of antibiotic at which no growth could be seen. The tolerance level of each strain was calculated using the formula: Tolerance=MBC/MIC (May et al., 1998).

### Growth Kinetic Analysis and Viability Assay

Growth kinetics of *B. subtilis* ATCC 6633 and *S. aureus* ATCC 25923 were assayed by growing the bacteria in 9ml LB or MHB2, respectively, at 37°C and 200rpm. One hour after the inoculum, bacterial cultures were added with 1ml of NP-TEICO or NP-VANCO (at 4mg/ml, corresponding to 615μg/ml of loaded teicoplanin for NP-TEICO, or 840μg/ml of conjugated vancomycin for NP-VANCO), or with nonconjugated teicoplanin or vancomycin (supplemented at the concentrations of the corresponding nanoconjugated antibiotics loaded onto IONPs). Growth was monitored by measuring the OD_600nm_ at regular time intervals, up to 5h from the inoculum, when cultures reached the stationary phase of growth. At this point, 10μl of undiluted or serially diluted bacterial cultures was plated on MHA. Plates were incubated at 37°C and after 24h, viable counts (expressed as colony forming units per ml, CFU/ml) were estimated.

### Fluorescence Microscopy Analysis

The LIVE/DEAD BacLight fluorescence assay (L7007, Molecular probes; ThermoFisher Scientific, Monza, Italy) was employed to investigate the effect of nonconjugated and nanoconjugated GPAs on *B. subtilis* ATCC 6633 and *S. aureus* ATCC 25923 cells, following the manufacturer’s instructions. After cultivation overnight at 37°C and 200rpm, bacterial cultures were diluted at OD_600nm_=0.1 in 9ml LB or MHB2 and incubated as above with 1ml of nanoconjugated antibiotics (at a concentration of 4mg/ml), or with an amount of nonconjugated teicoplanin or vancomycin equal to that loaded onto the corresponding IONPs (615μg/ml for teicoplanin, 840μg/ml for vancomycin). After 5h, each bacterial solution was centrifuged at 3,300 *×g* for 15min, followed by re-suspension of the pellets in sterile saline solution (0.9% *w*/*v* NaCl). Samples were incubated at room temperature for 1h with mixing every 15min, then washed twice with saline solution. Pellets were finally re-suspended in 1ml of saline solution and 3μl of dye mixture was added, then incubated in dark for 15min. Fluorescence images were acquired with an optical microscope with appropriate filters (Axiophot; Carl Zeiss, Milan, Italy), by trapping 5μl of stained bacteria between a slide and a cover lip. Total fluorescence intensity of bacteria was quantified with the free open-source software ImageJ (National Institute of Health, Unites States; Schneider et al., 2012). Intensities were indicated as % relative to the saturation fluorescence within the field. Red and green fluorescence stains corresponded to live or dead bacteria, respectively (Arakha et al., 2015).

### Biofilm Assay

Overnight cultures of *S. aureus* ATCC 6538P, *S. aureus* ATCC 25923, and *S. aureus* ATCC 43300 were diluted 1:100 in fresh TSB medium and 1ml of the diluted culture was added to each well of a 24-well polystyrene plate. On the basis of the previous experience on nanoconjugated teicoplanin inhibiting biofilm formation in *S. aureus* ATCC 6538P (Armenia et al., 2018), nonconjugated or nanoconjugated antibiotics were administered at concentrations corresponding to at least 4-fold the antibiotic MICs. An equivalent amount of IONPs was used as control. Cells were incubated at 37°C for 24h (for *S. aureus* ATCC 6538P and ATCC 43300) or for 48h (*S. aureus* ATCC 25923) and the inhibition of biofilm formation was evaluated as previously described (Armenia et al., 2018). Briefly, planktonic biomass was removed and collected, and wells were washed once with 1ml of PBS. Adherent biomass was quantified by crystal violet (CV) staining as follows: 1ml of 0.1% *w*/*v* CV was added to each well for approximately 20min to stain the biofilm, after which CV was removed, and each well was washed twice with 1ml PBS. The remaining CV, indicating the amount of biofilm, was dissolved in acetic acid 33% *v*/*v* for 10min and spectrophotometrically measured at 590nm. In order to evaluate the effect of different treatments on cellular viability, the planktonic phase was axenically collected, adherent cells were recovered by carefully scraping them and suspended in 1ml of PBS by mild pipetting. Viable counts – expressed as CFU per ml (CFU/ml) in cell suspensions and as CFU per well (CFU/well) in adherent biomass – were estimated using a plate count technique. A volume (10μl) of undiluted or serially diluted samples was plated onto LB agar plates and incubated for 24h at 37°C. Three replicates for each condition were analyzed within one experiment, and each experiment was repeated at least three times, using independent cultures.

### Confocal Analyses of Biofilm

Overnight *S. aureus* ATCC 6538P cultures were diluted 1:100 in TSB medium. A sample of 3ml of diluted culture was added to a glass coverslip placed on the bottom of 35mm diameter Petri dishes. Nonconjugated or nanoconjugated teicoplanin were added at 4μg/ml final concentration, and the corresponding concentration of IONPs was used as control. After 24h of incubation at 37°C, the planktonic phase was removed and adherent cells were stained with fluorochrome 4,4-difluoro-1,3,5,7-tetramethyl-8(2′methoxyphenyl)-4-bora-3a,4a-diaza-s-indacene at 5μM (Sunahara et al., 2007). To allow the penetration of the dye in the deepest layers of the biofilm, plates were incubated at 37°C for 30min, under agitation at 50rpm. Then, the coverslips were gently washed with PBS and transferred on microscope glass slides for the image acquisition through a 63× objective lens (Confocal light microscopy; Leica Microsystems, Wetzlar, Germany). Simulated 3D images were generated using ImageJ (Schneider et al., 2012).

### Application of an External Magnet Field to Biofilms

An external magnetic field can direct antibiotics conjugated to magnetic NPs to a specific site, as a biofilm-colonized surface, increasing local drug concentration and possibly improving the efficacy of the antibacterial treatment. To evaluate this aspect, overnight *S. aureus* ATCC 6538P cultures were diluted 1:100 in fresh TSB medium and 0.5ml of the culture was distributed in 24-well polystyrene plate. Nonconjugated and nanoconjugated teicoplanin (1μg/ml) and the corresponding concentration of IONPs were administered, and a cubic magnet (5×5×5mm) was positioned under each well. After 24h of bacterial growth at 37°C, planktonic cells were removed, and the adherent biomass was quantified by CV staining as previously described.

### Cytotoxicity Test and Macrophage Uptake of NPs

*In vitro* cytotoxicity of NP-TEICO and NP-VANCO was estimated on the tumor cell line SKOV-3 from ovary adenocarcinoma, cultured as previously reported (Cappellini et al., 2015; Armenia et al., 2018). Cell cytotoxicity was determined measuring ATP content with the RealTime-Glo™ MT Cell Viability Assay (Promega, Milan, Italy), according to the manufacturer’s instructions. Five hundred cells were plated in a 96-well plate with 200μl of RPMI-1640 cell medium. After 24h, cells were exposed to increasing concentrations of nanoconjugated or nonconjugated GPAs, or to an equivalent concentration of IONPs. A 2× solution of the substrate and NanoLuc^®^ Enzyme was added, followed by cell incubation at 37°C in 5% CO_2_-humidified atmosphere. Luminescence was read every 24h using the Infinite F200 plate reader (Tecan Group, Männedorf, Switzerland).

For the phagocytosis assay, the human promonocytic THP-1 cell line (Scaldaferri et al., 2018) was cultured in RPMI-1640 medium added with 10% v/v FBS and 1% w/v glutamine, and maintained in a humidified incubator (37°C, 5% CO_2_). THP-1 cells, at a concentration of 10^5^ cells/ml, were differentiated into M0 macrophages by the addition of 5ng/ml PMA for 48h as reported by Scaldaferri et al. (2018). Differentiation was performed on glass coverslips (12mm Ø) placed on the bottom of 12-well assay plates. Cells were then incubated for 24h with 5 and 50μg/ml IONPs, NP-TEICO, and NP-VANCO and visualized by Prussian Blue staining for iron detection. Cells were fixed in ice-cold ethanol for 5min, stained with an equal volume of 2% v/v hydrochloric acid and 2% w/v potassium ferrocyanide trihydrate for 15min. Samples were then washed with distilled water and dried by increasing concentrations of ethanol, then mounted in DePeX (Serva, Heidelberg, Germany). Observations were performed with a Zeiss Axiophot microscope under bright light illumination and photographs were acquired by a Zeiss AxioCam ERc5s camera (Carl Zeiss, Milan, Italy).

### Statistics

All experiments were repeated at least three times. Mean and standard deviation (SD) were calculated using Microsoft Excel 2003 (Microsoft Corporation, Redmond, United States). Data were analyzed by a one-way ANOVA (Origin_7.0 SR0; Origin lab Corporation, Northampton, United States). Significant effects of treatments were estimated (*p*<0.05, *p*<0.01, and *p*<0.0001).

## Results

### Synthesis and Physical Characterization of Nanoconjugated GPAs

**Figure 1:**
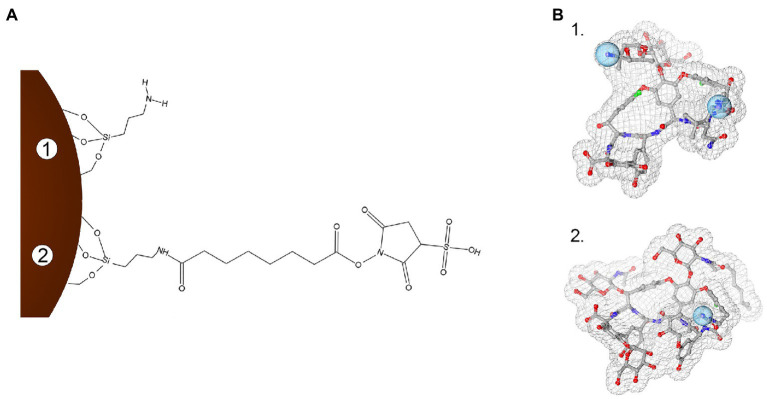
Schematization of the conjugation chemistry used to link teicoplanin and vancomycin onto iron oxide nanoparticles (IONPs; not in scale). Panel **A**: Surface modification steps with APTES functionalization (1) and subsequent BS3 linker binding (2). Panel **B**: 3D molecular structures of vancomycin (1, PDB entry 1FVM) and teicoplanin (2, PDB entry 3VFK). Blue spheres indicate amine groups used for conjugating GPAs to BS3 moiety. In (**B**), GPA structures were rendered with 3D ProteinImager (Tomasello et al., 2020).

Iron oxide nanoparticles were synthetized by the co-precipitation method, using the protocol by Balzaretti et al. (2017) optimized by Armenia et al. (2018). Conjugation of GPAs to NP-APTES was conducted by using the homobifunctional cross-linker BS3, which contains an amine-reactive N-hydroxysulfosuccinimide (NHS) ester at each end of an 8-carbon spacer arm. The NHS esters reacted with the amines present in the antibiotic molecules (one reactive amino group in teicoplanin and two in vancomycin), thus forming stable amidic bonds (Figure 1).

Conjugation of NPs with GPAs was followed by FTIR-ATR, as shown in Supplementary Figure S1. Spectra of IONPs showed the bands characteristic for the Fe-O bond; the functionalization with APTES and the conjugation with teicoplanin or vancomycin introduced specific changes in the corresponding IR spectra, indicating the formation of covalent bonds linking antibiotics to IONPs (Supplementary Figure S1). TEM micrographs (Supplementary Figures S2A,E) showed that IONPs had a spherical shape and an average diameter of 9.3±2.6nm, with good stability and no tendency to aggregation. Diameter was not influenced by the functionalization of IONPs with APTES (diameter of NP-APTES=9.2±3.1nm; Supplementary Figures S2B,F) and was only slightly increased by conjugating GPAs (Supplementary Figures S2C,D,G,H). Both NP-TEICO and NP-VANCO presented spherical shapes, with an average diameter of 9.9±3.2nm and 10.3±3.6nm, respectively.

Dynamic light scattering (DLS) was employed to measure the hydrodynamic size of the different NPs (Supplementary Table S1). Diameter and average size distribution (PDI) of IONPs and NP-APTES were in line with previous results (Armenia et al., 2018). IONPs presented a slight polydispersity, typical for NPs synthesized by co-precipitation method (Wu et al., 2015) and an average diameter of 14.5±0.7nm. The addition of the APTES coating to the NP core determined a rise in the hydrodynamic diameter (25.8±0.7nm), but the most evident increase was observed after conjugation of the two GPAs (380.0±2.0nm for NP-TEICO, 628.0±1.5nm for NP-VANCO). As previously observed (De Palma et al., 2007; Gonçalves et al., 2017), NP sizes estimated by DLS are significantly different from those obtained by TEM observations. This phenomenon is generally associated to the formation of additional hydrate layers in aqueous solutions due to antibiotic shells around NPs, which are not enough electron dense to be detected at the electron microscope. Additionally, a partial aggregation of the conjugated NPs in the experimental conditions used for DLS analysis might contribute to such an increase in hydrodynamic sizes (Szpak et al., 2013). Although teicoplanin and vancomycin are both glycosylated heptapeptides, the hydrodynamic size of NP-VANCO significantly exceeded the one of NP-TEICO; this could be probably explained by the more hydrophobic nature of teicoplanin due to the lipid tail present in teicoplanin and absent in vancomycin (Treviño et al., 2014; Marcone et al., 2018).

The measurement of ζ-potential (Supplementary Table S1) confirmed the correct coating of IONPs: indeed, a more positive net charge was observed, as a consequence of the presence of the positive amino groups of APTES on the particle surface. The additional conjugation of the GPAs led to a further increase in ζ-potential, which guaranties the colloidal stability of the nano-GPA systems.

Different combinations of GPA and BS3 linker concentrations were then used to optimize the antibiotic binding to NP-APTES (data not shown). Under the best reaction conditions so far identified (for teicoplanin: 4mg/ml of NP-APTES in 10mM borate buffer pH 8.2, 22.3μg/ml BS3, 1mg/ml teicoplanin; for vancomycin: an equal amount of NP-APTES in the same buffer, 44.6μg/ml BS3, 2mg/ml vancomycin), 155±14μg of teicoplanin and 210±40μg of vancomycin were loaded on average per mg of IONPs (data achieved after a dozen of different preparations for each GPA), corresponding to an average of 61.5 and 42% conjugation yield for teicoplanin and vancomycin, respectively. The chemical stability of the so-prepared NP-TEICO and NP-VANCO was tested at -20, 4, and 25°C. Measuring the release of teicoplanin and vancomycin by spectrophotometric analysis of the incubation buffer, *ca*. 25 and 30% of teicoplanin and vancomycin, respectively, were released after the first week of conservation. After that, the nanoconjugated antibiotic preparations remained considerably stable: after 4weeks, approximately 60% of teicoplanin and *ca*. 70% of vancomycin were still bound to IONPs.

### Antimicrobial Activity of Nanoconjugated GPAs

Antibacterial activity of the two nanoformulations was tested by agar diffusion assay towards two Gram-positive bacteria (*S. aureus* ATCC 25923 and *B. subtilis* ATCC 6633) and one Gram-negative bacterium (*E. coli* ATCC 35218). As expected for GPAs, both NP-TEICO and NP-VANCO were able to inhibit the growth of Gram-positives (Supplementary Figure S3), while no inhibition was observed for *E. coli* (data not shown). Notably, no inhibition halos were formed by the equivalent concentration of IONPs (Supplementary Figure S3), confirming that the antimicrobial activity of NP-TEICO and NP-VANCO is due to the conjugated GPAs and not to the NPs themselves. After 4weeks from their preparation, NP-TEICO and NP-VANCO maintained 70 and 55%, respectively, of their initial antimicrobial activity, consistently with the data on the chemical stability, reported above. Under the same conditions, water solutions of teicoplanin and vancomycin retained *ca*. 90% of their initial antimicrobial activity.

Table 1 reports the MICs and the MBCs of nonconjugated and nanoconjugated teicoplanin and vancomycin towards a collection of clinically relevant microorganisms, including *S. aureus*, *S. epidermidis*, *S. haemolyticus*, and *E. faecalis* strains. MICs of nonconjugated teicoplanin and vancomycin were comparable against *B. subtilis*, MSSA, MRSA, and *S. epidermidis* strains, which were all susceptible to both antibiotics [i.e., with MIC ≤2μg/ml, as defined by the Clinical and Laboratory Standards Institute (CLSI, 2018)]. *E. faecalis* ATCC 29212 and VanB-type *E. faecalis* ATCC 51299 were susceptible to teicoplanin, being on the contrary intermediate-resistant (i.e., MIC between 4 and 8μg/ml) or resistant (i.e., MIC ≥16μg/ml), respectively, to vancomycin. *S. haemolyticus* 3902 was resistant to teicoplanin and susceptible to vancomycin, as previously reported (Biavasco et al., 2000). MICs and MBCs of NP-TEICO towards all these susceptible and resistant strains followed the same trends as those measured for the nonconjugated antibiotic, confirming that nanoconjugated teicoplanin conserved the same spectrum of action, with only a slight reduction in potency. Instead, the potency reduction after antibiotic conjugation was more evident in the case of NP-VANCO, when compared to the activity of the corresponding nonconjugated antibiotic. Indeed, some Gram-positives (i.e., *B. subtilis*, MSSA ATCC 6538P, MRSA, *S. haemolyticus*, and *S. epidermidis*), susceptible to free vancomycin, were intermediately resistant towards NP-VANCO. As expected, nanoconjugated and nonconjugated GPAs were inactive toward VanA-type *E. faecalis* 9160188401-EF-34 (Van Bambeke, 2006; Binda et al., 2014) and the Gram-negative *E. coli*.

**Table 1:** Minimum inhibitory concentrations (MICs), minimal bactericidal concentrations (MBCs), and tolerance levels of nonconjugated and nanoconjugated teicoplanin and vancomycin.

	MIC (μg/ml)				MBC (μg/ml)				Tolerance level			
	TEICO	NP-TEICO	VANCO	NP-VANCO	TEICO	NP-TEICO	VANCO	NP-VANCO	TEICO	NP-TEICO	VANCO	NP-VANCO
*B. subtilis* ATCC 6633	2	0.5	2	4	>128	>128	>128	>128	>64	>128	>64	>32
*S. aureus* ATCC 6538P (MSSA)	1	2	1	4	128	128	>128	>128	128	64	>128	>32
*S. aureus* ATCC 25923 (MSSA)	0.25	0.5	0.25	1	16	64	32	>128	64	128	128	>128
*S. aureus* ATCC 43300 (MRSA)	0.5	1	1	4	64	>128	>128	>128	128	>128	>128	>32
*S. haemolyticus* 3902	16	32	2	8	64	>128	16	128	4	>4	8	16
*S. epidermidis* strain 4	2	4	2	8	64	128	32	>128	32	32	16	>16
*E. faecalis* ATCC 29212	0.5	0.5	4	8	32	64	>128	>128	64	128	>32	>16
*E. faecalis* ATCC 51299 (VanB)	0.5	1	16	32	64	>128	>128	>128	128	>128	>8	>4
*E. faecalis* 9160188401-EF-34 (VanA)	>128	>128	>128	>128	>128	>128	>128	>128	–	–	–	–
*E. coli* ATCC 35218	>128	>128	>128	>128	>128	>128	>128	>128	–	–	–	–

The values represent the average of the data from three independent experiments.

### Effects of Nanoconjugated GPAs on the Bacterial Growth Kinetics and Cell Viability

**Figure 2:**
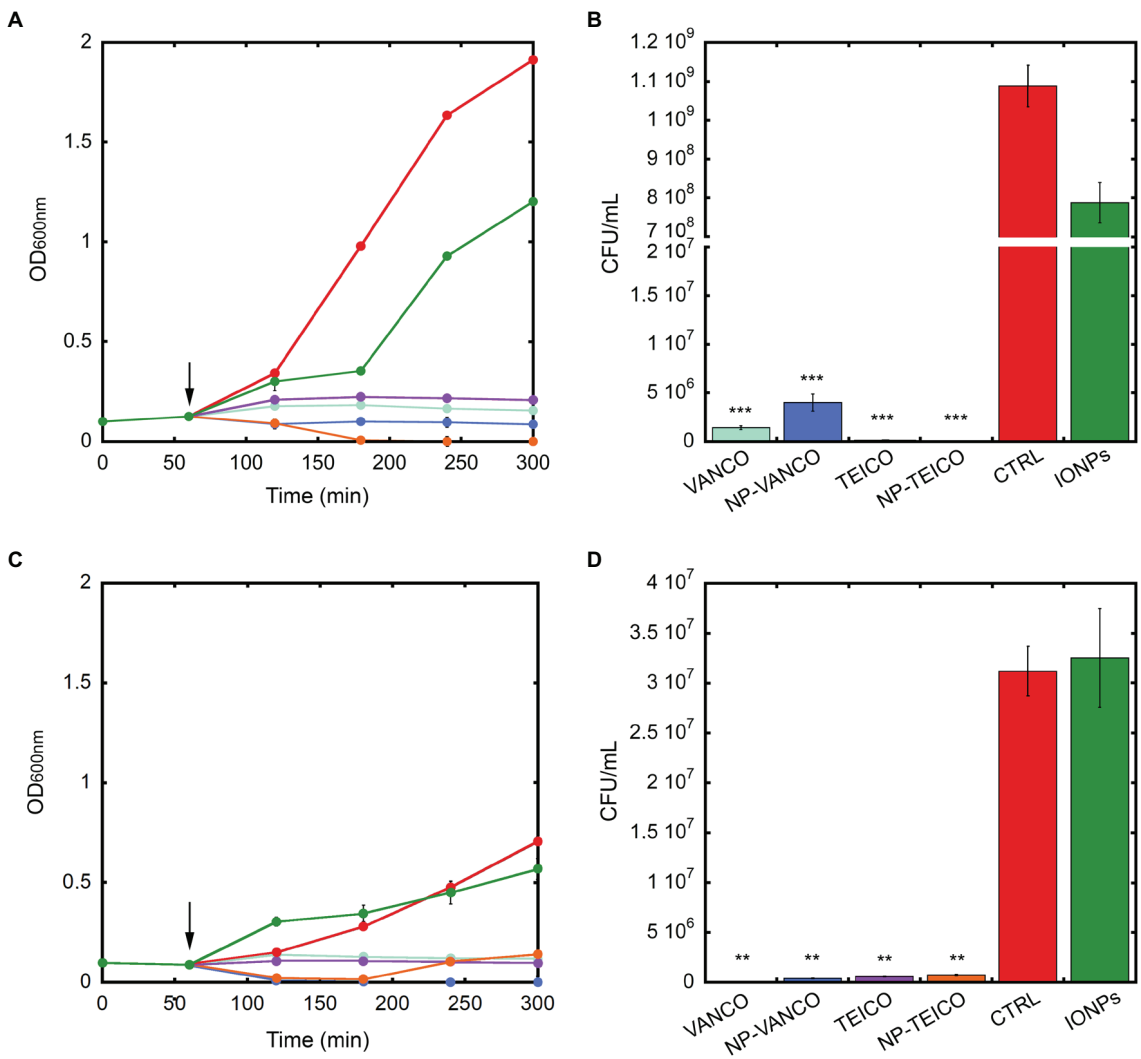
Population growth kinetics of *B. subtilis* ATCC 6633 (panel **A**) and *S. aureus* ATCC 25923 (panel **C**) exposed to 0.4mg/ml of IONPs (green), NP-VANCO (blue, corresponding to 84μg/ml of conjugated vancomycin), NP-TEICO (orange, corresponding to 61.5μg/ml of conjugated teicoplanin), or to nonconjugated teicoplanin (TEICO, violet) and vancomycin (VANCO, turquoise), supplemented at the concentrations of the corresponding nanoconjugated antibiotics loaded onto IONPs. Cultures without any addition (CTRL, red) were used as control. Black arrows indicate the addition (after 1h) of IONPs, or nanoconjugated, or nonconjugated antibiotics to bacterial populations. In panels **B** and **D**, bacterial cell viability of *B. subtilis* ATCC 6633 and *S. aureus* ATCC 25923, respectively, measured as CFU/ml after 5-h growth. Experiments were conducted in triplicate for each condition. Statistical analyses were performed by one-way ANOVA (^**^p<0.01; ^***^p<0.0001).

The effect of the two nanoconjugated GPAs on the growth and viability of representative Gram-positive bacteria was further investigated. Equal concentrations of nonconjugated or nanoconjugated teicoplanin or vancomycin were added at the log phase during the growth kinetics of *S. aureus* ATCC 25923 and *B. subtilis* ATCC 6633. Cultures without any addition or supplemented with an equivalent amount of IONPs were used as controls. In both the bacteria, the addition of nonconjugated and nanoconjugated GPAs prevented the bacterial population to enter into the exponential growth phase. The extent of the growth inhibition effect was comparable among teicoplanin, vancomycin, NP-TEICO, and NP-VANCO (Figures 2A,C). Indeed, the addition of IONPs determined particularly for *B. subtilis* a slight reduction in the normal growth rate, probably due to a transient species-specific electrostatic interaction occurring between bacterial cells and IONPs, as previously reported by other authors (Ebrahiminezhad et al., 2014; Arakha et al., 2015; Dinali et al., 2017; Armenia et al., 2018; Shkodenko et al., 2020). Consistently, CFU measurements at the end of the experiment clearly showed that exposing *B. subtilis* and *S. aureus* to nonconjugated or nanoconjugated antibiotics caused an almost complete clearance of bacterial populations, thus confirming the antibiotic activity of nanoconjugated GPAs against Gram-positives (Figures 2B,D). The effect of IONPs was also in this case slightly different between the two microorganisms: in the case of *S. aureus* ATCC 25923 no bactericidal effect of IONPs was observed, as cell survival percentage was equal to that of the untreated population (Figure 2D). A reduction in cell viability was, instead, observed after exposure of *B. subtilis* ATCC 6633 to IONPs (Figure 2B), although this difference was not statistically significant and not comparable to the clear bactericidal activity of nanoconjugated and nonconjugated GPAs.

The LIVE/DEAD BacLight fluorescence assay (Supplementary Figure S4) confirmed the bactericidal effect of nanoconjugated antibiotics on *S. aureus* and *B. subtilis*. In this assay, viable cells are stained green by the Syto9 fluorescence dye, whereas nonviable cells are stained red by propidium iodide fluorescence dye (Arakha et al., 2015). Untreated cells of both *S. aureus* ATCC 25923 and *B. subtilis* ATCC 6633 (Supplementary Figures S4A,B) were stained green by Syto9 dye, indicating the presence of 85% viable cells for *B. subtilis* and 98% for *S. aureus*. Instead, cells turned red after exposure to both nonconjugated or nanoconjugated teicoplanin (Supplementary Figures S4C–F) and vancomycin (Supplementary Figures S4G–J), thus confirming the bactericidal effect of GPAs in both forms. Interestingly, when NP-TEICO or NP-VANCO was present, bacteria tended to aggregate on them (Supplementary Figures S4E,F,I,J), further favouring the clearance of bacterial populations.

### Effect of Nanoconjugated GPAs on *Staphylococcus aureus* Biofilms

**Figure 3:**
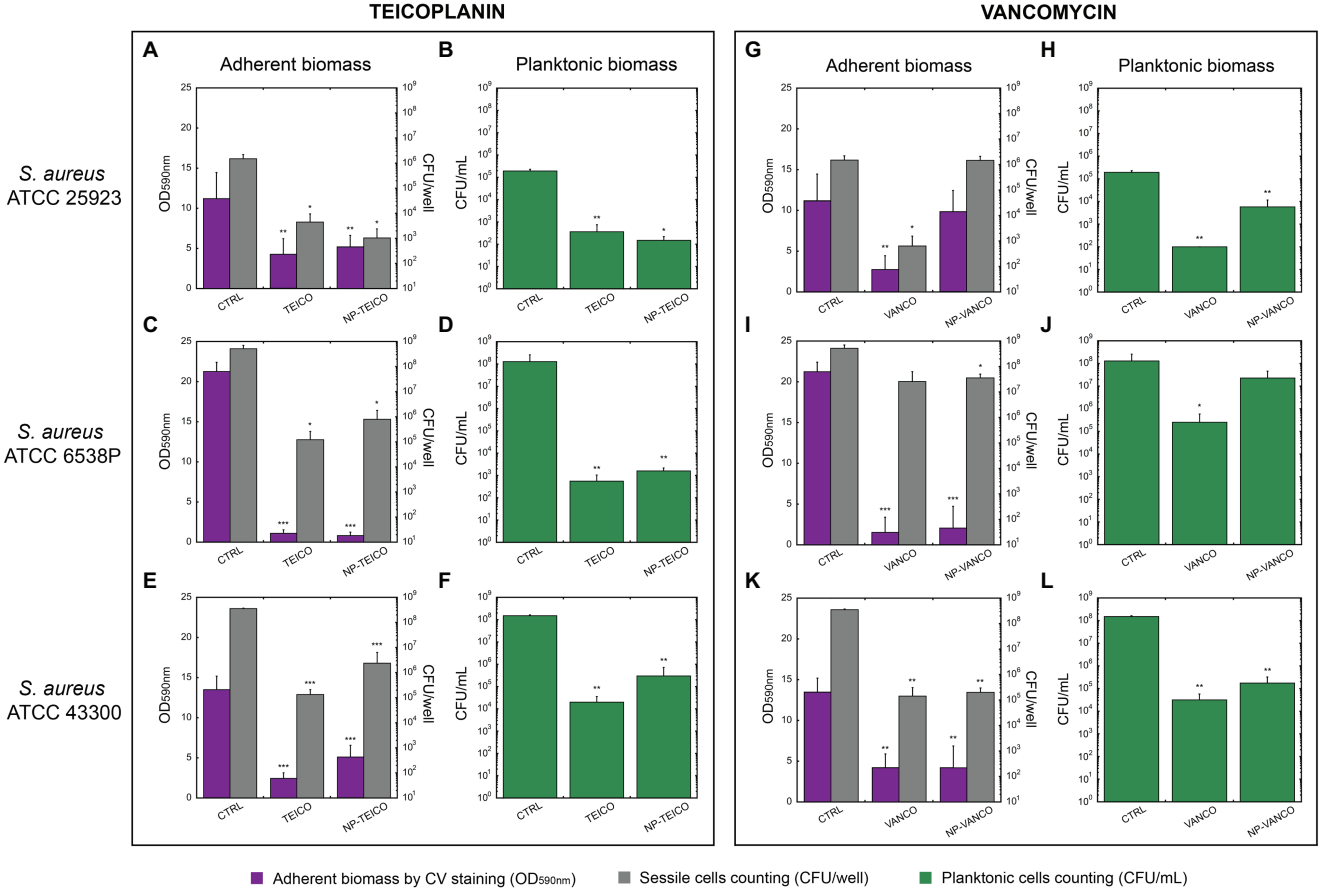
Inhibition of biofilm formation in three *S. aureus* strains by nonconjugated and nanoconjugated teicoplanin or vancomycin. In all panels, ‘CTRL’ refers to the untreated control. Panels **A** and **B**: effect of nonconjugated or nanoconjugated teicoplanin at 1μg/ml on biofilm formation by *S. aureus* ATCC 25923 grown for 48h. Panels **C** and **D**: effect of nonconjugated or nanoconjugated teicoplanin at 4μg/ml on biofilm formation by *S. aureus* ATCC 6538P grown for 24h. Panels **E** and **F**: effect of nonconjugated or nanoconjugated teicoplanin at 8μg/ml on biofilm formation by *S. aureus* ATCC 43300 grown for 24h. Panels **G** and **H**: effect of nanoconjugated and nonconjugated vancomycin at 1μg/ml on biofilm formation by *S. aureus* ATCC 25923 grown for 48h. Panels **I** and **J**: effect of nanoconjugated and nonconjugated vancomycin at 4μg/ml on biofilm formation by *S. aureus* ATCC 6538P grown for 24h. Panels **K** and **L**: effect of nanoconjugated and nonconjugated vancomycin at 16μg/ml on biofilm formation by *S. aureus* ATCC 43300 grown for 24h. Adherent biomass is estimated spectrophotometrically at 590nm after crystal violet staining (violet bars in **A**,**C**,**E**,**G**,**I**,**K**) and by bacterial cell counting of sessile cells expressed as CFU/well (grey bars in **A**,**C**,**E**,**G**,**I**,**K**). Cellular concentration (CFU/ml) of planktonic population was also evaluated (green bars in **B**,**D**,**F**,**H**,**J**,**L**). The values are the means of at least three independent experiments and the bars represent standard deviations. Statistical analyses were performed by one-way ANOVA (^*^p<0.05; ^**^p<0.01; ^***^p<0.0001).

NP-TEICO, NP-VANCO and their nonconjugated GPAs were tested on *S. aureus* biofilms (Figure 3) using three different strains of *S. aureus* (Table 1) – i.e., two methicillin sensitive *S. aureus* strains (ATCC 25923 and ATCC 6538P) and one resistant strain (ATCC 43300) – known from the literature for producing biofilms at different extent (Rose and Poppens, 2009; Artini et al., 2012). The effect of IONPs *per se* was ruled out by preliminary experiments (Supplementary Table S2), in which different concentrations of IONPs were tested on planktonic and adherent subpopulations of the biofilms formed by the three *S. aureus* strains. None statistically significant inhibitory activity on biofilm production and cell viability by IONPs was revealed in comparison to untreated controls (Supplementary Table S2).

*S. aureus* ATCC 25923 was the most sensitive strain among the three, to both teicoplanin and vancomycin (MIC=0.25μg/ml, Table 1). Nanoconjugated and nonconjugated antibiotics were tested at concentrations corresponding to four-fold the MIC, i.e., 1μg/ml. As shown in Figure 3A, NP-TEICO inhibited biofilm formation at a similar extent to teicoplanin: a decrease of ~60% in adherent biomass was detected, compared to the untreated sample. Furthermore, both formulations of teicoplanin caused a significant decrease (~3 Log unit) in cell count of both adherent and planktonic cells (Figures 3A,B). Conversely, NP-VANCO was less active than vancomycin on both adherent and planktonic populations (Figures 3G,H). Additionally, NP-VANCO (Figures 3G,H) was also less effective than the corresponding preparation of NP-TEICO (Figures 3A,B).

*S. aureus* ATCC 6538P was a better biofilm former than *S. aureus* ATCC 25923: it formed in 24h an adherent biomass two-fold more abundant if compared to the one produced by ATCC 25923 strain in 48h of growth (CTRL bars in Figures 3C,D,I,J). As reported in Table 1, *S. aureus* ATCC 6538P was also less susceptible than *S. aureus* ATCC 25923 to both teicoplanin and vancomycin (MIC=1μg/ml, Table 1). Formulations of nonconjugated and nanoconjugated teicoplanin (Figures 3C,D) and vancomycin (Figures 3I,J) were thus administered at a concentration of 4μg/ml, four-fold the MIC. In the case of NP-TEICO, its effect was similar to teicoplanin and caused a significant inhibition of biofilm formation: a ~95% decrease in adherent biomass and a ~3 Log unit reduction of sessile cell counting was observed (Figure 3C). A 5 Log unit decrease in planktonic cell concentration was observed under treatment with both nonconjugated and nanoconjugated teicoplanin (Figure 3D). In the case of NP-VANCO, the effect was similar to vancomycin on biofilm adherent biomass (Figure 3I), which was inhibited of almost 90% by both nonconjugated and nanoconjugated vancomycin. The two formulations differed in their activity vs. the planktonic cells: cell count of vancomycin-treated planktonic cells was significantly lower (~3 Log units) than in untreated samples, but NP-VANCO activity on planktonic cells was lowered in comparison to nonconjugated vancomycin (Figure 3J). Vancomycin *per se* (Figures 3I,J) was indeed less active than teicoplanin (Figures 3C,D) especially on planktonic cell population.

Finally, the methicillin-resistant *S. aureus* ATCC 43300 strain (MRSA) was found to form less biofilm than ATCC 6538P strain (CTRL bars in Figures 3E,F,K,L), but its biofilm was particularly tolerant to antibiotics. At 2μg/ml teicoplanin concentration (four-fold the MIC of 0.5μg/ml, Table 1), none significant inhibitory effect on biofilm formation was observed (data not shown) and it was necessary to administer 8μg/ml of teicoplanin, a concentration 16-fold higher than the MIC, to get an inhibitory effect. At this concentration, teicoplanin inhibited biofilm formation causing a ~80% decrease in adherent biomass and ~3 Log unit decrease in its cell viability as compared to untreated cells. The inhibitory effect of NP-TEICO was slightly reduced in comparison with teicoplanin, causing a reduction of ~65% of adherent biomass and ~2 Log unit decrease in adherent cell viability (Figure 3E). Reductions of ~4 and ~3 Log units were observed in suspended cell counts, following the treatment with teicoplanin and NP-TEICO, respectively (Figure 3F). Nonconjugated and nanoconjugated vancomycin were tested at an equivalent concentration of 16-fold higher than the MIC (=1μg/ml, Table 1). The biofilm formation of the strain was inhibited in a similar manner by 16μg/ml of vancomycin and by the corresponding NP-VANCO preparation: the adherent biomass decreased of about 70% and CFUs/well of about 3 Log units (Figure 3K). Considering the planktonic population, 4 and 3 Log unit reductions were observed in cell counting for vancomycin and NP-VANCO treated samples, respectively (Figure 3L).

### Nanoconjugated Teicoplanin to Counteract *Staphylococcus aureus* Biofilm Formation

**Figure 4:**
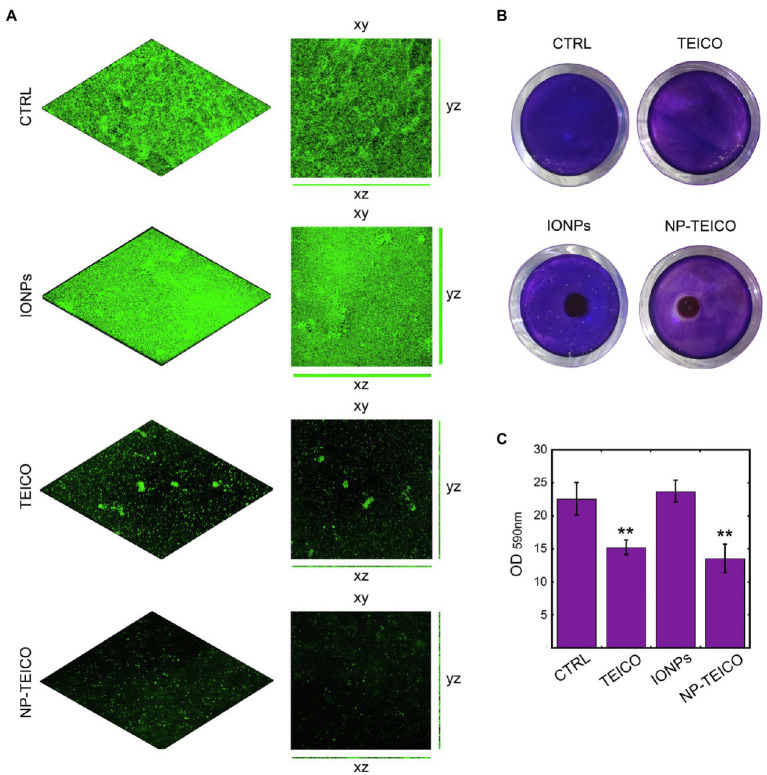
Effect of nonconjugated and nanoconjugated teicoplanin on *S. aureus* ATCC 6538P biofilm on different surfaces. Panel **A**: CLSM images of *S. aureus* ATCC 6538P biofilm on glass surface, grown for 24h without any addition (CTRL) or in the presence of nonconjugated (TEICO) or nanoconjugated (NP-TEICO) teicoplanin at 4μg/ml, or with an equivalent amount of IONPs. Panel **B**: *S. aureus* ATCC 6538P biofilm formation in 24-well polystyrene plate, exposed to a magnet (5×5×5mm) localized under each well. The bacteria were grown 24h on the plastic surface in different conditions: without any treatment (CTRL), incubated with nonconjugated teicoplanin (TEICO) or with nanoconjugated teicoplanin (NP-TEICO) added in both cases at 1μg/ml, or with an equivalent amount of IONPs. Panel **C**: Adherent biomass formation in 24-well polystyrene plate after 24h of bacterial growth at 37°C, quantified spectrophotometrically at 590nm after crystal violet staining. Statistical analyses were performed by one-way ANOVA (^**^p<0.01).

Since the above results on the effect of nanoconjugated GPAs on *S. aureus* biofilm formation indicated the better performance of NP-TEICO in comparison to NP-VANCO, further investigations were dedicated to studying the biofilm formation in the presence of nanoconjugated teicoplanin by confocal microscope analyses. The better producer of biofilm biomass, *S. aureus* ATCC 6538P, was selected for these experiments. Bacterial cells were treated with 4μg/ml of nonconjugated or nanoconjugated teicoplanin and biofilm development was allowed on glass surfaces for 24h at 37°C. Then, cells and biofilm matrix became visible thanks to the fluorophore administration (Figure 4A). The addition of IONPs determined the formation of a biofilm more homogeneous than the control one. The addition of teicoplanin and of NP-TEICO prevented the formation of a well-structured biofilm, if compared to the controls: under nonconjugated and nanoconjugated antibiotic treatment, only few fluorescent cells were observed and no matrix was visible (Figure 4A).

Considering that the advantage of using NP-TEICO might consist in directing magnetic nanoconjugated antibiotic towards the area of interest using an external magnetic field, we mimicked the hypothetical treatment of a contaminated surface attracting NP-TEICO on a small area (the center of a well in a 24-well polystyrene plate) to evaluate its effect on *S. aureus* 6538P biofilm formation (Figure 4B). Teicoplanin administered at the concentration of 1μg/ml inhibited by *ca*. 34% the biofilm formation when compared to untreated *S. aureus* (Figures 4B,C). The treatment of bacteria with NP-TEICO, inhibited of *ca*. 43% the biofilm formation with respect to IONPs (Figures 4B,C). The application of an external magnet field attracted the nanoparticles at the center of the well and a clear inhibition halo in the crystal violet staining indicated the localized inhibitory effect around NP-TEICO (Figure 4B).

### Cytotoxicity and Macrophage Phagocytosis of Nanoconjugated GPAs

**Figure 5:**
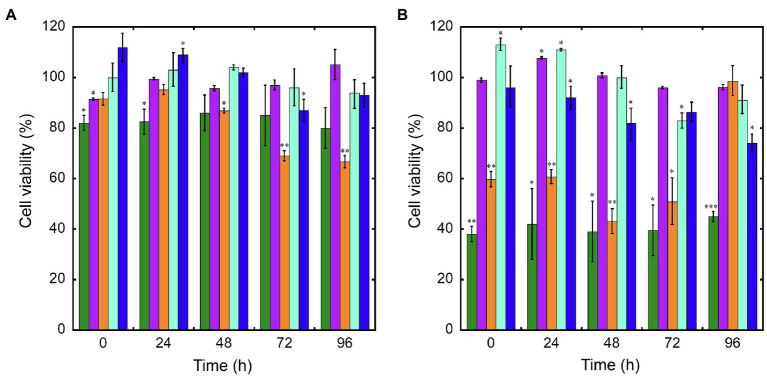
Cell viability of SKOV-3 after exposure up to 96h to IONPs (green bars), teicoplanin (violet), NP-TEICO (orange), vancomycin (turquoise), or NP-VANCO (blue). Cell viability was expressed as a percentage of viable cells compared to the untreated sample, set as 100%. The amount of nonconjugated or nanoconjugated GPA to be added was as follows: 0.9μg/ml (panel **A**) and 9μg/ml (panel **B**). In the case of nanoGPAs and IONPs, the amounts to be added were defined considering the antibiotic loaded on IONPs under the conditions described in Material and Methods. The values are expressed as mean±standard deviation of three independent experiments. One-way ANOVA analysis, ^*^p<0.05; ^**^p<0.01; ^***^p<0.0001.

**Figure 6:**
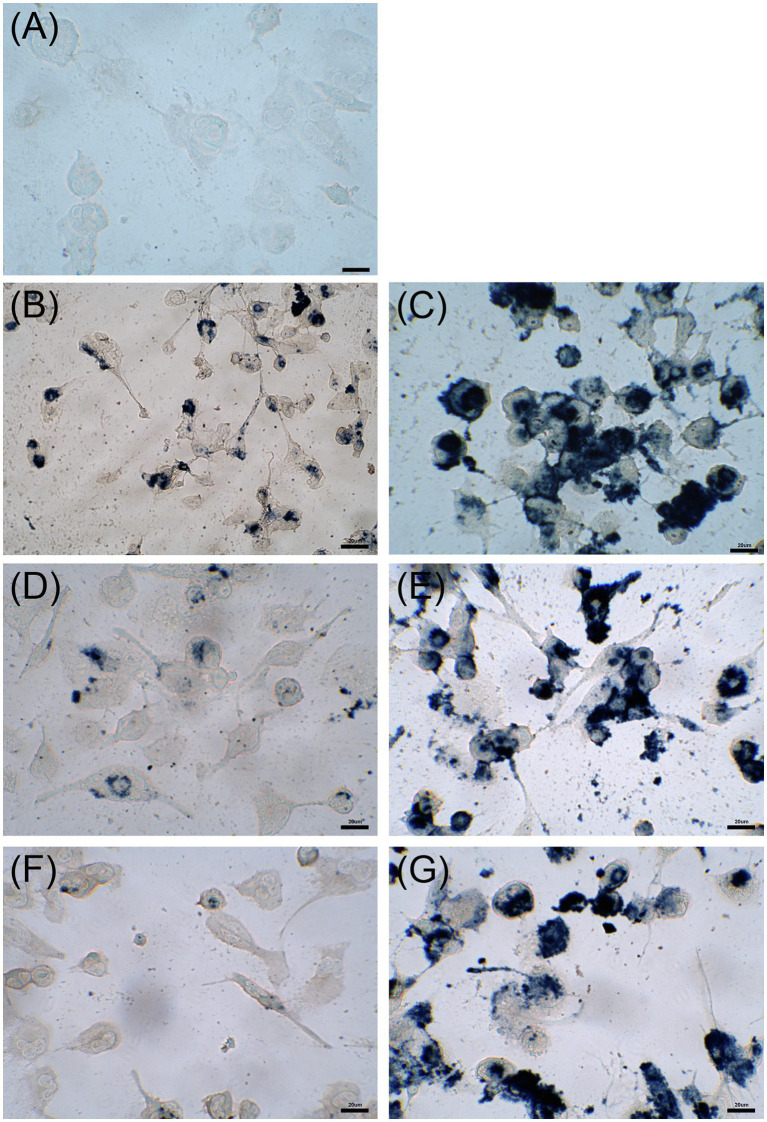
Uptake by THP-1 cells after 24h of incubation with 5μg/ml of IONPs (panel **B**), 50μg/ml of IONPs (panel **C**), 5μg/ml of NP-TEICO (panel **D**), 50μg/ml of NP-TEICO (panel **E**), 5μg/ml of NP-VANCO (panel **F**), 50μg/ml of NP-VANCO (panel **G**) and control untreated cells (panel **A**). Prussian Blue staining of NP ferric ions was observed at the optical microscope; bars are 20μm.

Cytotoxicity of nanoconjugated and nonconjugated GPAs was investigated using the well-established immortalized tumor cell line SKOV-3 (Cappellini et al., 2015). Both teicoplanin and vancomycin are antibiotics without any detectable cytotoxicity effect, also when tested at nearly 100-fold their MICs on susceptible bacteria. Indeed, SKOV-3 cells responded to exposure to IONPs, NP-TEICO and NP-VANCO – tested at concentrations in the range of antibacterial MICs – in a dose-dependent manner (Figure 5). As reported previously (Bava et al., 2013; Armenia et al., 2018), naked IONPs themselves exerted a cytotoxic effect: at 5μg/ml (Figure 5A), cell viability was reduced of about 20%, in comparison to untreated cells, independently from exposure time, whereas at nearly 50μg/ml (Figure 5B), naked IONPs reduced cell viability by more than 60% (after 24h of exposure) to 50% (after 96h). In the case of NP-TEICO, when the antibiotic was supplemented at a concentration in the range of antibacterial MICs towards susceptible bacteria (0.9μg/ml; Figure 5A), cell viability was reduced by no more than 33% (after 96h of exposure). At a concentration 10 times higher (9μg/ml; Figure 5B), cell viability was decreased from 39% (after 24h) to 1.4% (after 96h). For NP-VANCO, at 0.9μg/ml no significant decrease in cell viability was observed at 96h (Figure 5A), whereas at 9μg/ml the reduction in cell viability was no more than 17% (after 96h of exposure; Figure 5B). All together these data confirm what already reported by other authors, i.e., the coverage of IONPs with nontoxic molecules tends to reduce their intrinsic cytotoxicity and improve their biocompatibility in the range of antimicrobial effective concentrations (Xiang et al., 2017; Armenia et al., 2018; Xie et al., 2018). On the other hand, no difference was observed in the macrophage uptake of NP-TEICO, NP-VANCO, or of their naked nanocarriers, assayed using the Prussian Blue staining (Figure 6). The characteristic blue color developing from the reaction of potassium ferrocyanide with NP ferric ions highlighted a comparable macrophage dose-dependent uptake of nanoconjugated antibiotics or of their nanocarriers in 24h. That macrophages engulfed IONPs, NP-TEICO, and NP-VANCO in a comparable mode confirmed what was already reported by other authors, i.e., that when macrophages are exposed *in vitro* to nanomaterials, they usually engulf them (Qie et al., 2016; Verçoza et al., 2019).

## Discussion

In our previous paper (Armenia et al., 2018), we demonstrated that NP-TEICO systems could be prepared and used against Gram-positive pathogens. In that case, teicoplanin (but not vancomycin) was conjugated to APTES-functionalized IONPs, using standard EDC/NHS (N-(3-dimethylaminopropyl)-N′-ethylcarbodiimide hydrochloride/N-hydroxysuccinimide) chemistry. In this work, we have introduced the homobifunctional cross-linker BS3, with the goal to improve GPA loading on IONPs thanks to the protruding arms of the linker and the reactivity of the free amino groups of the GPAs (one in teicoplanin and two in vancomycin). With this reaction, we improved the conjugation efficiency. Indeed, in this work, we have loaded more than 150μg of teicoplanin on average per mg of IONPs instead of the nearly 100μg (Armenia et al., 2018). Additionally, in this paper, we applied the new conjugation reaction to vancomycin, which was linked to IONPs at a concentration higher than 200μg for mg of IONPs. It is worth noting that in our previous trials using EDC/NHS chemistry with vancomycin, we never obtained stable NP-VANCO preparations (unpublished results), although in the literature it is reported that the carboxylic group of vancomycin could be used, similarly to that of teicoplanin, for the binding to NPs (Kell et al., 2008; Zhu et al., 2015; Chen et al., 2019). It is anyhow true that the comparison of the loading capacity of the different preparations is quite challenging due to the great variability in size, geometry, crystalline structure, and capping polymers used with IONPs (Kell et al., 2008; Wu et al., 2015; Wang et al., 2019). Importantly, our superparamagnetic NP-TEICO and NP-VANCO, with an average diameter of approximately 10nm, were chemically stable (slightly more NP-TEICO than NP-VANCO) and could be stored at different temperatures.

### Antimicrobial Activity of GPA-NP Systems

While nanoconjugation partially reduced NP-VANCO antimicrobial activity in comparison to nonconjugated vancomycin, the prepared NP-TEICO maintained the typical GPA antimicrobial spectrum of activity towards Gram-positive bacteria, including clinical isolates and resistant strains (Van Bambeke, 2006; Binda et al., 2014; Yushchuk et al., 2020b). NP-TEICO was more active than NP-VANCO on VanB-resistant enterococci, NP-VANCO was more active than NP-TEICO on *S. haemolyticus*, whereas both preparations were not active against VanA enterococci and the Gram-negative representative *E. coli*. Although many authors have previously reported that IONPs, as well as other types of metal oxide NPs, possess an intrinsic antimicrobial activity (Gabrielyan et al., 2019; Das et al., 2020; Shkodenko et al., 2020), in the different experiments hereby conducted (i.e., agar diffusion assay, BacLight fluorescence assay, bacterial growth kinetics, CFU measurement) a significant bacteriostatic and bactericidal activity was conferred to IONPs only following their conjugation with teicoplanin and vancomycin. As previously reported (Ebrahiminezhad et al., 2014; Arakha et al., 2015; Dinali et al., 2017; Armenia et al., 2018), naked IONPs can interact mainly *via* electrostatic interactions with bacterial cell envelopes, arresting transiently cell growth and, in sporadic cases, causing cell death, but this phenomenon greatly differs in magnitude from the potent and selective antimicrobial action of ‘last-resort’ antibiotics such as GPAs towards Gram-positive bacterial strains. In a recent review, Shkodenko et al. (2020) reported that in general naked IONPs have to be used at mg/l concentrations to show some antibacterial activity.

### Antibiofilm Activity of GPA-NP Systems

Although some published reports indicated that IONPs exert an inhibitory activity on biofilms formed by food associated bacteria (Al-Shabib et al., 2018) or by *S. aureus*, *E. coli*, and *Pseudomonas aeruginosa* growing on different polymeric surfaces (Thukkaram et al., 2014), our results on *S. aureus* biofilm clearly demonstrated that at the concentrations at which nonconjugated and nanoconjugated GPAs are active, IONPs *per se* did not exert any appreciable inhibitory effect. Similarly to gold and silver NPs, IONPs might exert an inhibitory effect on biofilm-embedded cells by causing a mechanical disruption of the biofilm matrix (Li et al., 2019) and by intercepting those non-specific interactions (electrostatic, hydrophobic, hydrogen-bonding, ionic, and van der Waal interactions) that are responsible for the adhesion of biofilm-forming bacteria to surfaces (Joshi et al., 2020). These effects, however, become evident at relatively high concentrations of IONPs (Joshi et al., 2020; Shkodenko et al., 2020). As reported by Al-Shabib et al. (2018), IONPs can inhibit production of alginate, which is a key component of the extracellular polymeric matrix in *P. aeruginosa* biofilm, or of other exopolysaccharides that maintain the biofilm architecture and act as protective barrier. Biofilm structure and composition are complex and dramatically differ from species to species and even among strains belonging to same species, as in the case of *S. aureus* (Rose and Poppens, 2009; Artini et al., 2012). Thus, it is not surprising that the responses to NP-based formulations are variable, since they might depend on particular combinations of different factors such as biofilm maturity and surface composition on the one side, and chemistry, nanoparticle size, surface charge, surface chemistry, and NP concentration on the other side (Arias et al., 2018; Joshi et al., 2020).

Since the early 1960s, vancomycin has been the first-choice drug for treating patients infected by invasive MRSA, but its use is currently limited by slow antibacterial speed, low tissue penetration, and increasing drug resistance. Moreover, penetration of vancomycin into viable *S. aureus* biofilms analyzed by confocal scanning laser microscopy was found extremely low (Jefferson et al., 2005). Although teicoplanin offers several advantages over vancomycin, such as a longer half-life, better stability *in vivo*, lower nephrotoxicity and ototoxicity, and activity on VanB-phenotype vancomycin-resistant pathogens, its action on biofilm formation has not been adequately investigated, yet. Our results showed that teicoplanin and, albeit at a less extent, vancomycin could reduce biofilm formation by the three different strains of *S. aureus* used in this study, two MSSA and one MRSA. The relative impact of the two GPAs differed on the three strains, the MRSA being the most resistant as reported by other authors (Fulaz et al., 2020; Gao et al., 2020). The nanoconjugated GPAs maintained the same activities as the two nonconjugated counterparts, being NP-TEICO more active than NP-VANCO. Teicoplanin differs from vancomycin for its lipid chain that confers it a superior antimicrobial potency. The lipid chain better anchors teicoplanin to the membrane/cell wall interface where the antibiotic binds to the d-alanyl-d-alanine terminus of the peptidoglycan precursors, arresting cell wall biosynthesis (Treviño et al., 2014; Yushchuk et al., 2020a). It would be interesting to investigate further if the more hydrophobic nature of teicoplanin in comparison to vancomycin might explain its improved activity on biofilm formation.

### Potential Advantage of GPA-NP Systems

Advantages of using NP-TEICO instead of the soluble teicoplanin counterpart are due to the magnetic properties of the carrying IONPs that can be directed by an external magnetic field to a specific site, as a biofilm-colonized surface, increasing the local drug concentration. We showed that NP-TEICO could be attracted by an external magnet and that it drastically reduced biofilm formation around its concentration zone. The concept can be extended to targeting a deep soft tissue infection, or reaching infected tissues or organs, reducing the need of using elevated doses to treat resistant bacteria and concomitantly limiting toxic side effects. Although further investigations are needed to develop adequate experimental systems and animal models to study the antimicrobial and anti-biofilm potential of NP-TEICO highlighted in this paper, we think that these lines of evidence pave the way for a promising use of NP-TEICO and more in general of IONPs carrying other novel GPAs. This approach can also be extended in future for the controlled and targeted use of other last-resort potent molecules such as daptomycin and polymyxins. The targeting therapy concept originates from and is widely used in cancer therapy (Naskar and Kim, 2019; Ajinkya et al., 2020) but the times are mature, due to a dramatic increase in AMR, to shift it to the anti-infective therapeutic area.

### Nanotoxicity of GPA-NP Systems

A still unsolved issue of using IONP-carried antibiotics remains assessing their intrinsic level of cytotoxicity, which could eventually limit their further *in vivo* utilization (Wu et al., 2015; Arias et al., 2018). Although IONPs are considered the most biocompatible among metal oxide NPs and many clinical applications based on their use *in vivo* have been already proposed (i.e., magnetic resonance imaging, thermal ablation therapy, treatment of iron-deficient anemia; Arias et al., 2018; Ajinkya et al., 2020; Wang et al., 2020), the knowledge on their interaction with animal cells and models is still very limited (Naskar and Kim, 2019; Das et al., 2020). Herein, we report that the cytotoxic effect of IONPs to mammalian cell lines *in vitro*, when used in the range of the antibacterial MICs of GPAs, is relatively low and it seems to be mitigated by the coverage with non-toxic molecules such as GPAs themselves. Nevertheless, a complete assessment of nanomaterial toxicity requires considering their administration routes, distribution and stability inside the different body districts, factors that can be investigated only through *in vivo* studies.

Another correlated aspect that needs to be further investigated *in vivo* is how rapidly and efficiently host phagocytes, if recruited, might engulf nanoantibiotics. We hereby reported that *in vitro* cultures of macrophages engulfed both bare and antibiotic-conjugated IONPs. This is not surprising since several studies have shown that macrophages internalize NPs (Verçoza et al., 2019), although, in principle, their surface modifications might allow evasion of phagocytic clearance (Qie et al., 2016). In any case, little is known about the *in vivo* recruitment of macrophages by nanomaterials and, *a fortiori*, by nanoantibiotics, depending again on their administration routes, body district distribution and physicochemical properties of their surface. Cappellini et al. (2015) evaluated the biodistribution of a nanoenzyme system *in vivo* 24-h post-treatment, and reported that intravenously injected IONPs were found mainly in the cytoplasm of Kupffer cells and spleen histocytes, and no macrophage recruitment was detectable in heart, testis, and brain. Bonfanti et al. (2020) observed that *Xenopus laevis* embryos accumulated IONPs in their gut (IONPs were found dispersed within the enterocytes of a well-preserved intestinal epithelium), without causing any acute toxicity nor teratogenicity.

### Future Perspectives

Infection animal models are urgently needed for better evaluating the potential of nanoconjugated antibiotics for topic, oral, or systemic use, including their stability toward the proteolytic activity and to the different pHs occurring at sites of infection. To the best of our knowledge, there is only one study on an IONP-conjugated GPA tested in an infection animal model. This is the case of the recent work of Chen et al. (2019), who reported the effect of IONPs carrying vancomycin on *C. difficile* in mice infection model, demonstrating that nanoconjugated vancomycin exerted a therapeutic effect higher than free vancomycin, reducing intestinal inflammation, facilitating mucosal viability, and limiting the antibiotic side effects on intestinal microbiota. Our next goal is to test NP-TEICO and NP-VANCO in the invertebrate infection model that we have recently developed for comparing GPA efficacy in curing *in vivo* infections (Montali et al., 2020). We employed easy-to-handle larvae of the silkworm *Bombyx mori* infected by *S. aureus*. Due to its great advantages (i.e., safe handling, low rearing costs, low antibiotic amount needed, no restrictions imposed by ethical and regulatory issues), this silkworm infection model could help in rapidly solving pending issues about *in vivo* efficacy and toxicity of nanoconjugated antibiotics. Only few papers reported on testing nanomaterials in insect models (Thomaz et al., 2020; Moya-Andérico et al., 2021). Indeed, they might be useful to gain a better understanding of the physiological responses of living organisms to nanomaterials and to speed up their development towards clinical applications.

## Concluding Remarks

In conclusion, in this paper we described novel nanoformulations of teicoplanin and vancomycin covalently conjugated to IONPs. These nanoformulations were chemically stable and especially NP-TEICO conserved the typical spectrum of antimicrobial activity of the nonconjugated glycopeptide antibiotic. NP-TEICO and, although to a lesser extent, NP-VANCO were effective in reducing *S. aureus* biofilm formation. In particular, when concentrated by the action of an external magnetic field, NP-TEICO exerted a localized inhibitory effect on biofilm formation even at a very low antibiotic concentration. Finally, we proved that the conjugation of GPAs to IONPs reduced their intrinsic cytotoxicity toward a human cell line, thus paving the way for their study in animal models.

## Data Availability Statement

The original contributions presented in the study are included in the article/Supplementary Material, further inquiries can be directed to the corresponding author.

## Author Contributions

FM, RG, FB, and GB conceived the experiments, interpreted the results, and wrote the manuscript. IA and FG developed and produced the NP-conjugated antibiotics. FB conducted and interpreted the experiments on the microbiological activity of NPs. VO and EM conducted and interpreted the experiments on biofilms. RG conducted and interpreted the experiments on human cell line. All authors contributed to the article and approved the submitted version.

## Funding

This work was supported by the University of Insubria grant “Fondo di Ateneo per la Ricerca” 2018 and 2019 to FM and VO, and by the HOTZYMES project (EU’s Horizon 2020 Programme, FET OPEN, grant agreement no. 829162) to IA, RG, and GB.

## Conflict of Interest

The authors declare that the research was conducted in the absence of any commercial or financial relationships that could be construed as a potential conflict of interest.

## Publisher’s Note

All claims expressed in this article are solely those of the authors and do not necessarily represent those of their affiliated organizations, or those of the publisher, the editors and the reviewers. Any product that may be evaluated in this article, or claim that may be made by its manufacturer, is not guaranteed or endorsed by the publisher.
