# Supplementary material for: Antimicrobial Activity of Nanoconjugated Glycopeptide Antibiotics and Their Effect on Staphylococcus aureus Biofilm
Source: Front Microbiol. 2021 Dec 2;12:657431. doi: 10.3389/fmicb.2021.657431 (PMC8674785; doi:10.3389/fmicb.2021.657431)
Supplement: Supplementary file 1 [file Table_1.DOCX]

Supplementary Material

**Table 1S**. Physical parameters of synthesized NPs.

|  | **Polydispersity** | **Diameter (nm)** | **ζ-potential (mV)** |
| --- | --- | --- | --- |
| IONPs | 0.13 | 14.5 ± 0.7 | 11.0 ± 0.6 |
| NP-APTES | 0.18 | 25.8 ± 0.7 | 22.5 ± 0.5 |
| NP-TEICO | 0.20 | 380.0 ± 2.0 | 32.4 ± 0.9 |
| NP-VANCO | 0.21 | 628.0 ± 1.5 | 25.5 ± 0.3 |

**Table 2S.** Effect of IONPs at different concentrations on the biofilms produced by the three different *Staphylococcus aureus* strains used in this work. Total biofilm adherent biomass (OD_590nm_), cellular viability of adherent population (CFU/well), and of planktonic cells (CFU/mL) were determined. The reported values are the means of at least three independent experiments ± standard deviation. Statistical analyses were performed by one-way ANOVA: for all strains, no significant difference between untreated samples and cells exposed to IONPs or NP-APTES was observed.

| ***S. aureus* strain** | **NP conc. (µg/mL)** | **Adherent biomass (OD_590nm_)** | | **Adherent biomass viability**  **(CFU/well)** | | **Planktonic biomass viability (CFU/mL)** | |
| --- | --- | --- | --- | --- | --- | --- | --- |
|  |  | **Untreated** | **IONPs** | **Untreated** | **IONPs** | **Untreated** | **IONPs** |
| ATCC 25923 | 8.4 | 11.26 ± 3.1 | 9.65 ± 2.75 | 1.09 x 10^6^ | 5.40 x 10^5^ | 1.30 x 10^5^ | 1.40 x 10^4^ |
| ATCC 6538P | 34.4 | 21.32 ± 1.09 | 18.86 ± 2.41 | 5.20 x 10^8^ | 3.50 x 10^8^ | 1.20 x 10^8^ | 1.70 x 10^8^ |
| ATCC 43300 | 82.6 | 13.55 ± 1.64 | 12.17 ± 0.64 | 3.60 ± 10^8^ | 1.80 x 10^8^ | 1.50 x 10^8^ | 1.55 x 10^8^ |

**
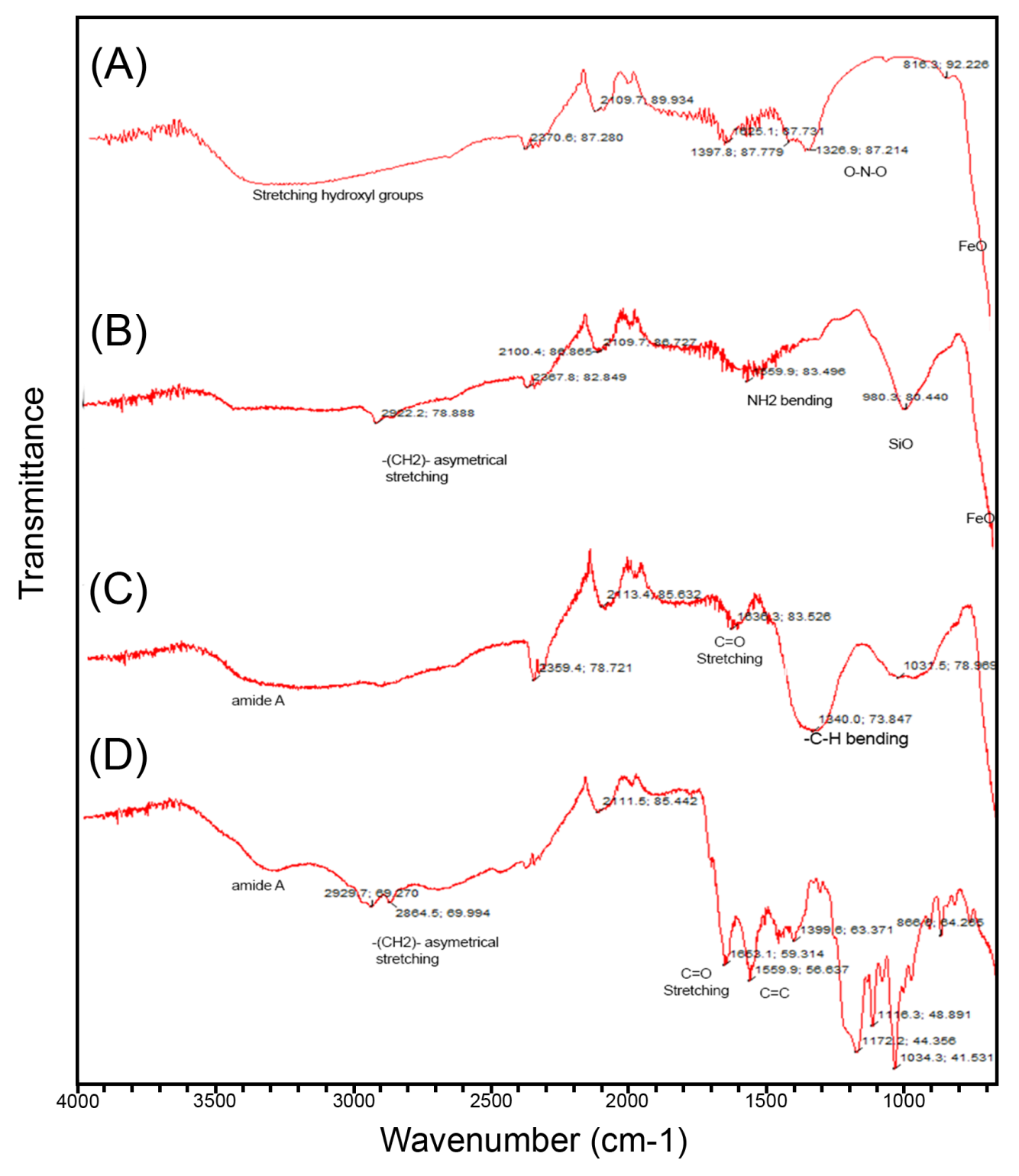
**

**Figure 1S.** Fourier Transform Infrared Spectroscopy in Attenuated Total Reflectance (FTIR-ATR) spectra of IONPs (**A**), NP-APTES (**B**), NP-TEICO (**C**), and NP-VANCO (**D**). The presence of a band peak about 400 cm^-1^ is characteristic for the Fe-O bond, present in all spectra (**A**,**B**,**C**,**D**). IONPs (**A**) showed a characteristic band between 3500 and 2500 cm^-1^ due to the stretching of the hydroxyl groups present on the NP surface. Functionalization with APTES (**B**) was demonstrated by the appearance of the following bands at: *i)* 890 cm^-1^ revealing the presence of silanols on NP surface (Villa et al., 2016; Yamaura et al., 2004), *ii)* 1600 cm^-1^, indicating the free amino groups introduced by APTES, and *iii)* 3000 cm^-1^ referred to the -CH bond. Conjugation of teicoplanin to IONPs (**C**) was confirmed by the rise of the bands at: *i)* around 3400 cm^-1^, which could be attributed to the stretching of the primary amine of the teicoplanin, *ii)* 1650 cm^-1^ from the C=O stretching, and *iii)* 1340 cm^-1^ due to the -C-H- bending present in the molecule (Coates, 2006; Kilinç et al., 2020). Binding of vancomycin to IONPs (**D**) was demonstrated by arising of the bands at 1650 and 1600 cm^-1^, indicating the presence of double bonds, respectively of the carbon-carbon and carbonyl bonds. The banding in the range 3500-2870 cm^-1^ results from the superposition of an amide band with -OH bonds. The vancomycin bond to IONPs (**D**) was also confirmed by the band at 1231 cm^-1^, due to the presence of an oxygen linked to two aromatic rings within the vancomycin molecule (Dinache et al., 2015), and by the absorption bands in the range 900-400 cm^-1^ that could be attributed to various bonds including-C-Cl.

**
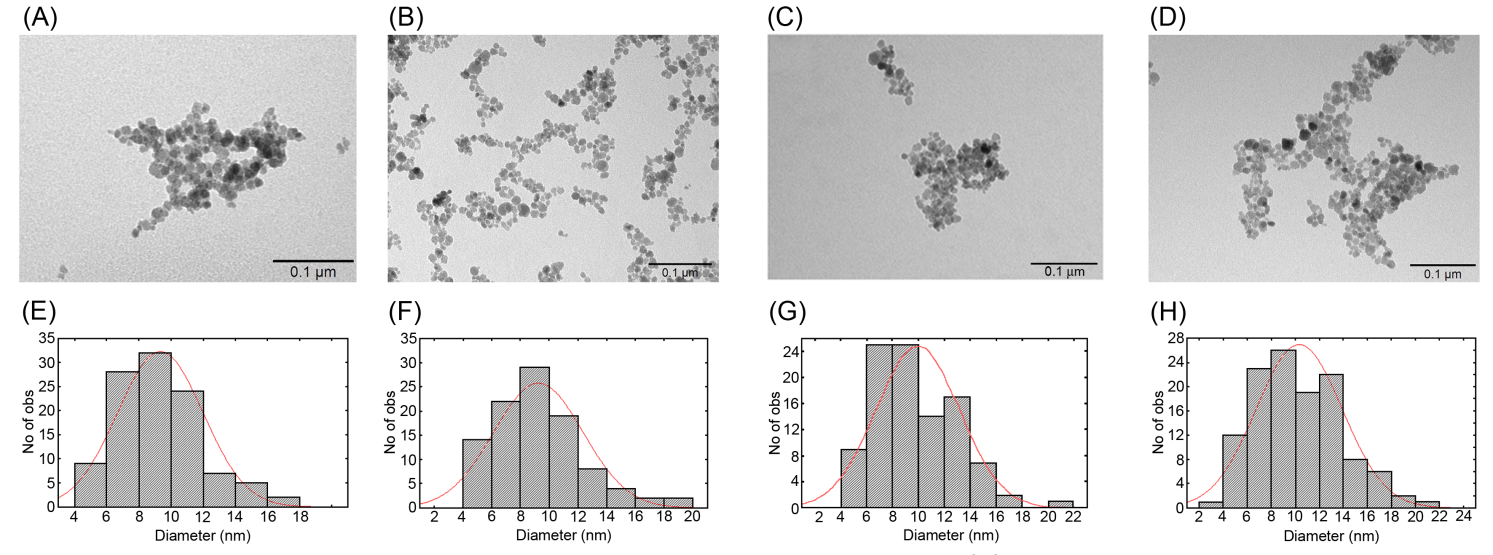
**

**Figure 2S**. TEM images and size distribution of IONPs (**A,E**), NP-APTES (**B,F**), NP-TEICO (**C,G**), and NP-VANCO (**D,H**).


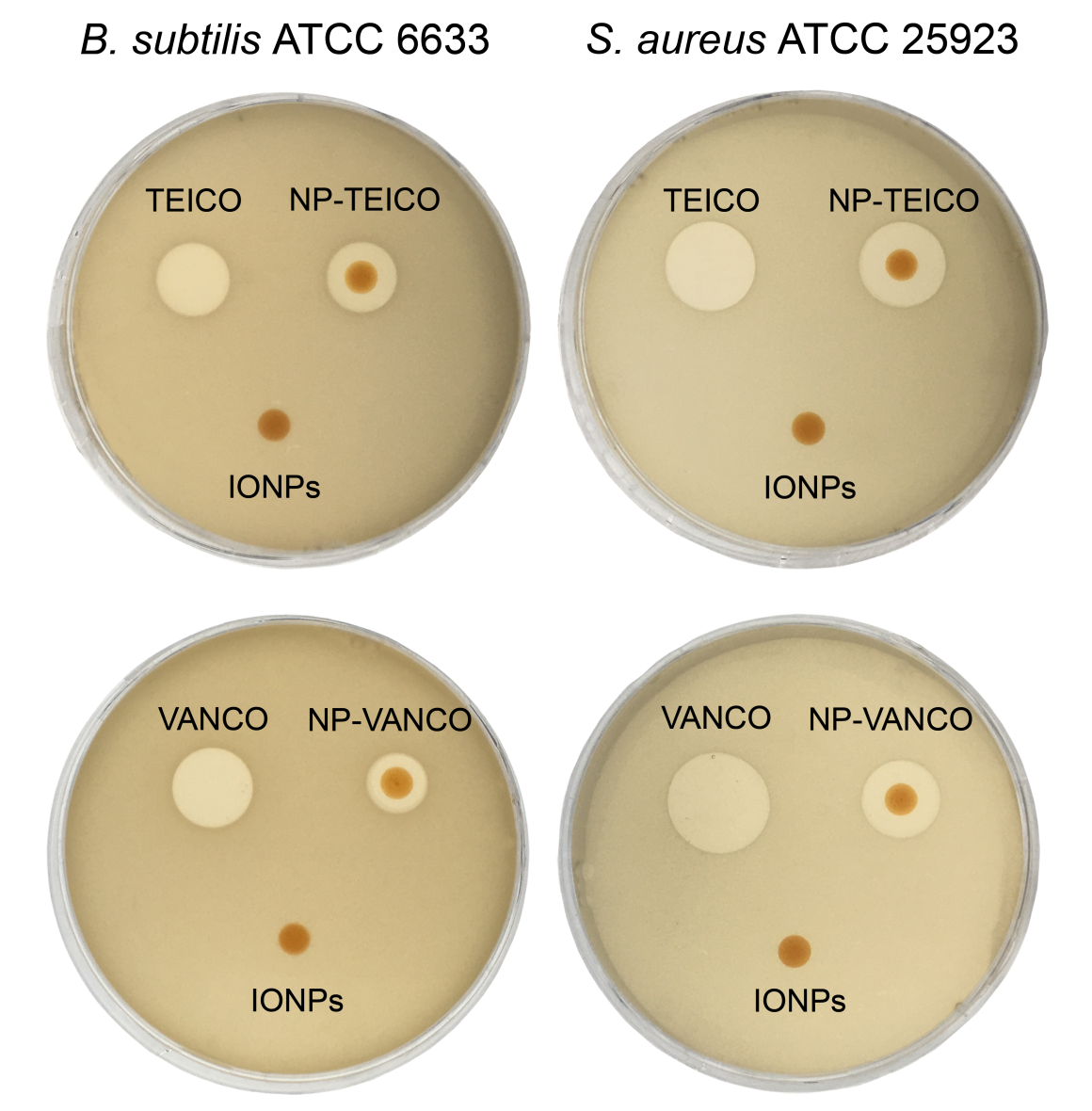


**Figure 3S**. Agar diffusion assay for estimating the antimicrobial activity of IONPs, NP-TEICO, NP-VANCO, and nonconjugated antibiotics (TEICO and VANCO) versus the Gram-positive bacteria *Bacillus subtilis* ATCC 6633 (on the left) and *S. aureus* ATCC 25923 (on the right). 10 μL of each sample was dropped onto the plate. NP preparations were added at 4 mg/mL concentration (equal to 615 μg/mL loaded teicoplanin for NP-TEICO, and 840 μg/mL vancomycin for NP-VANCO), whereas nonconjugated antibiotics were added at a concentration equal to that loaded on the corresponding IONPs. The fact that the sizes of inhibition halos were not perfectly comparable between nonconjugated and nanoconjugated antibiotics, is likely due to the lower diffusion rate of nanoantibiotics in agar medium.


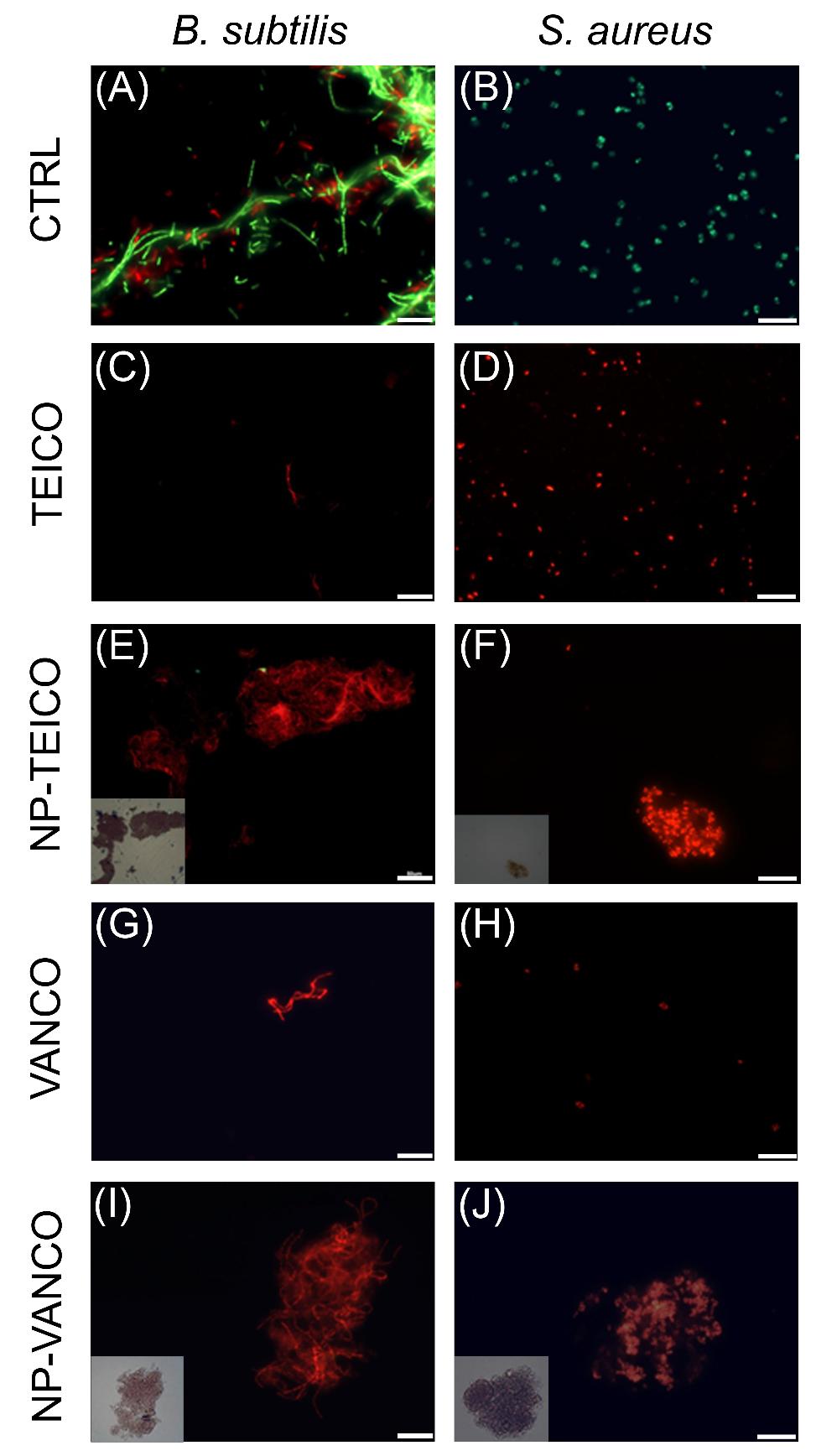


**Figure 4S**. Fluorescence microscopy images of live and dead cells of *B. subtilis* ATCC 6633 (column on the left) and *S. aureus* ATCC 25923 (column on the right) in the absence (control, CTRL) and presence of nonconjugated (TEICO and VANCO) and nanoconjugated teicoplanin and vancomycin (NP-TEICO and NP-VANCO). Scale bar: 12 µm.

**References**

Coates, J. (2006). “Interpretation of infrared spectra, a practical approach” in Encyclopedia of analytical chemistry: applications, theory and instrumentation, eds. R.A. Meyers and M.L. McKelvy (John Wiley & Sons Ltd), 10815-10837. doi: 10.1002/9780470027318.a5606.

Dinache, A., Boni, M., Alexandru, T., Radu, E., Stoicu, A., Andrei, I.R., et al. (2015). Surface properties of vancomycin after interaction with laser beams. *Colloids Surf. A Physicochem. Eng. Asp.*, 480, 328-335. doi: 10.1016/j.colsurfa.2014.08.023.

Kilinç, S., Pazarci, Ö., Çakmak, N.K., Taş, A. (2020). Does the addition of poly (glycolide‑co‑lactide) to teicoplanin‑containing poly (methyl methacrylate) beads change the elution characteristics? *Indian J. Orthop.* 54, 71-75. doi: 10.1007/s43465-020-00116-4.

Villa, S., Riani, P., Locardi, F., Canepa, F. (2016). Functionalization of Fe_3_O_4_ NPs by silanization: Use of amine (APTES) and thiol (MPTMS) silanes and their physical characterization. *Materials (Basel)* 9, 1-14. doi: 10.3390/ma9100826.

Yamaura, M., Camilo, R.L., Sampaio, L.C., Macêdo, M.A., Nakamura, M., Toma, H.E. (2004). Preparation and characterization of (3-aminopropyl)triethoxysilane-coated magnetite nanoparticles. *J. Magn. Magn. Mater.* 279, 210-217. doi:10.1016/j.jmmm.2004.01.094.
